# Neuroinflammatory changes of the normal brain tissue in cured mice following combined radiation and anti-PD-1 blockade therapy for glioma

**DOI:** 10.1038/s41598-021-84600-3

**Published:** 2021-03-03

**Authors:** Mariano Guardia Clausi, Alexander M. Stessin, Zirun Zhao, Stella E. Tsirka, Samuel Ryu

**Affiliations:** 1 Department of Radiation Oncology, Renaissance School of Medicine, Stony Brook University, Stony Brook, NY 11794 USA; 2 Department of Pharmacological Sciences, Renaissance School of Medicine, Stony Brook University, Stony Brook, NY 11794 USA

**Keywords:** Neurology, Oncology

## Abstract

The efficacy of combining radiation therapy with immune checkpoint inhibitor blockade to treat brain tumors is currently the subject of multiple investigations and holds significant therapeutic promise. However, the long-term effects of this combination therapy on the normal brain tissue are unknown. Here, we examined mice that were intracranially implanted with murine glioma cell line and became long-term survivors after treatment with a combination of 10 Gy cranial irradiation (RT) and anti-PD-1 checkpoint blockade (aPD-1). Post-mortem analysis of the cerebral hemisphere contralateral to tumor implantation showed complete abolishment of hippocampal neurogenesis, but neural stem cells were well preserved in subventricular zone. In addition, we observed a drastic reduction in the number of mature oligodendrocytes in the subcortical white matter. Importantly, this observation was evident specifically in the combined (RT + aPD-1) treatment group but not in the single treatment arm of either RT alone or aPD-1 alone. Elimination of microglia with a small molecule inhibitor of colony stimulated factor-1 receptor (PLX5622) prevented the loss of mature oligodendrocytes. These results identify for the first time a unique pattern of normal tissue changes in the brain secondary to combination treatment with radiotherapy and immunotherapy. The results also suggest a role for microglia as key mediators of the adverse treatment effect.

## Introduction

Recent success of immune checkpoint blockade as a cancer-treatment modality has led to increased long-term survival rates across different cancer patient populations. As such, long-term side effects of this treatment become an important subject of investigation, and to date there is a dearth of information available. Radiation therapy (RT) is a mainstay treatment for both primary and metastatic brain tumors, but unfortunately it carries a high risk of progressive cognitive decline. Putative mechanisms affecting cognition after RT include neuroinflammation, decline in neurogenesis, degradation of neuronal structure, vascular damage and alterations in the white matter integrity^1^. Several strategies have been evaluated to prevent or mitigate the development of late radiation cognitive impairment. In a previously published study, we reported the ability of the FDA approved drug fingolimod (FTY720) to increase tolerance of dentate gyrus neural stem cells (NSCs) in vitro and mitigate radiation-induced cognitive deficits^2^. Although the mechanism of radioprotection of fingolimod is unknown, it is reported to have immunomodulatory actions by preventing the egress of peripheral T lymphocytes from lymphoid tissues into the CNS^3^. Recent studies also demonstrated that it reduces microglial activation^4^. In another study, the use of PLX5622, a small molecule inhibitor of colony stimulated factor-1 receptor (CSF1R) which crosses the blood brain barrier, resulted in complete elimination of microglial cells and improvement in cognitive function following whole brain radiation^5^. These studies suggest that neuroinflammation has a major role in radiation-induced cognitive decline. The immune-mediated adverse effects become more crucial with the development of novel treatments combining brain-directed RT and immunotherapy. These treatments have shown efficacy against solid tumors by enhancing inflammation in the tumor microenvironment. In a recent clinical report, it was shown that patients with brain metastasis that received anti-PD-1 treatment after stereotactic radiosurgery presented MRI signals suggesting an exacerbation of the immunological response in the perilesional normal brain tissue. In fact, the histological examination of the small rim of normal tissue surrounding these lesions was characterized by infiltrating macrophages, myelin loss, reactive astrocytes, and hyalinization and sclerosis of blood vessels^6^.

We have previously established a model of glioblastoma in C57BL/6 mice with implantation of GL261 cells in the brain. After combination of whole brain RT (10 Gy single exposure) with anti-PD-1 immune checkpoint blockade treatment (RT + aPD-1), 75% of these mice become long-term survivors. The increased survival correlated with the tumor infiltration of CD8 + lymphocytes and peripheral macrophages and the polarization of microglia and macrophages towards a pro-inflammatory M1 phenotype^7^. In order to study the long-term cognitive effect of the treatments, we investigated the pathological changes in the normal brain tissue from mice that achieved complete tumor regression after RT + aPD-1 treatment and became long-term survivors. Specifically, we examined the infiltration of inflammatory cells and structural abnormalities in hippocampal neurogenesis and the subcortical white matter in the brain hemisphere contralateral to the tumor implantation. The combined RT + aPD-1 treatment produced long-lasting activation of microglial cells, complete abolishment of hippocampal neurogenesis, and decreased the number of oligodendrocytes in the subcortical white matter. Elimination of microglia with Plexxikon (PLX) 5622 didn’t restore hippocampal neurogenesis but prevented loss of mature oligodendrocytes, suggesting that these cells may act as mediators of the long-term adverse effects following RT + aPD1 treatment.

## Materials and methods

### Animal treatment

All animal studies were carried out in compliance with the ARRIVE guidelines. Immunocompetent C57BL/6 male mice were purchased from Charles River Breeding Lab (Wilmington, MA) and maintained on a 12:12 h light:dark cycle with food and water ad libitum*.* Our experimental model and protocol have been published^7^. For glioma implantation, the mice were anesthetized using ketamine (120 mg/kg) and xylazine (10 mg/kg). A midline incision was made on the scalp, and a small burr hole was drilled in the skull at stereotactic coordinates of bregma, − 1 mm anteroposterior and + 2 mm mediolateral. GL261-eGFP (30 × 10^3^) cells suspended in 1 µl of PBS were injected slowly over two minutes to the left frontal lobe of the brain at a depth of 3 mm. This tumor model system is well established in our and other laboratories^8–10^. The tumor reaches to the size of 2–3 mm about 10 days post implantation (dpi), and the median survival time is 25 days without any treatment. Sham-treated mice were injected with 0.15 M saline instead of tumor cells. For radiation therapy (RT), a single dose radiation of 10 Gy was delivered to the entire brain at 10 dpi by using 100 kVp animal irradiator (Phillips RT-100). This radiation dose was chosen for its therapeutic effect, being in the similar range with our prior clinical trial of SRT; it also corresponded to single exposure whole brain doses previously used by ours and other laboratories to study the normal tissue effects of cranial irradiation. Immune check point inhibitor was given with 10 mg/kg of aPD1, anti-mouse PD-1 antibody (clone RPM1-14, BioX Cell), by intraperitoneal injection immediately after RT at 10 dpi, and two more doses at 12 and 14 dpi.

To deplete the bone marrow-derived macrophage mice were treated with 300 µg of anti CSF1-R (clone AFS98, BioXCell) i.p. every other day starting from day 8 during the study duration^11^. To inhibit microglial infiltration PLX5622 was formulated in an AIN-76 rodent diet at a dose of 1200 mg/kg standard chow provided by PLX5622 Inc. The mice were fed standard chow until day 8, at which point they were switched to food containing the PLX5622 drug for the remainder of the experiment. To identify proliferating cells, 5-Bromo-2-deoxyuridine (BrdU, Sigma) was administered daily for 7 days at 50 mg/kg body weight intraperitoneally (i.p.) starting at 60 dpi.

The randomized treatment groups were: Sham + saline, Sham + aPD-1, Sham + RT, Sham + RT + aPD-1, Tumor + RT + aPD-1, Tumor + RT + aPD-1 + aCSF1R, and Tumor + RT + aPD-1 + PLX5622, with at least 6 mice per treatment arm. Only the cured mice that became long-term survivors were used for post-mortem analysis. Long-term tumor survivors are defined as RT + aPD-1 treated mice which lived over 90 dpi and pathological examination of the brain show no presence of tumor. To harvest the brain samples, mice were anesthetized with ketamine and xylazine before intracardiac perfusion with PBS followed by 4% PFA in PBS. The brains were removed and post-fixed in 4% PFA/PBS for 12 h followed by cryoprotection in 30% w/v sucrose for 48 h. All animal procedures were approved by the Stony Brook University Institutional Animal Care and Use Committee (IACUC). All methods were performed in accordance with the relevant guidelines and regulations.

### Immunohistochemistry

The extracted brains were embedded in optimal cutting temperature compound (Tissue-Tek) and sectioned using Leica cryostat. Coronal brain sections (20-μm thick) were collected for analysis. The following primary antibodies were used: anti-Iba1 (1.200, Wako Chemicals), anti-pCREB (1:400, Cell Signaling), anti-DCX (1:1000, Abcam), anti-GFAP (1:1500, Dako), anti-NeuN (1:200, Millipore), anti-BrdU (1:200, Abcam) anti-Olig2 (1:250, Abcam), anti-APC (CC1, 1:100, Millipore), and anti-MBP (1:1000, BioLegend). Secondary antibodies against the appropriate species were incubated for 2 h at room temperature (Jackson, West Grove, PA, USA). DAPI (4,6-Diamidino-2-phenylindole, 1 mg/ml, Sigma) was used for 15 min to counterstain nuclei.

### Histological analysis and cell quantification

Confocal images throughout the external capsule of the *corpus callosum*, the hippocampal dentate gyrus, the subventricular zone and the granule cell layer of the olfactory bulb were acquired at 40X magnification with a Leica TCS SP8X confocal microscope. Positive stained cells were counted using NIH Image J software in at least 6–10 sections per mice and the average results expressed as positive cells per field. For morphological analysis of microglia, confocal image stacks (zstacks) of cells positive for Iba1-immunostaining in the external capsule of the corpus callosum were collected with at 63 × magnification. Neurolucida software was used to trace the cell bodies and processes in order to create 3D reconstruction of the cells and Neurolucida explorer tool was used to analyze the cell shapes.

### Statistical analysis

For continuous outcomes, means were calculated and compared between experimental arms using ANOVA followed by Tukey’s post hoc test or Student’s *t*-test. Repeated ANOVA was used to analyze repeated measures over time. All data are presented as mean ± SEM of at least two independent experiments. *P* value less than 0.05 was considered as statistically significant.

## Results

### Radiation-induced acute alterations in hippocampal neurogenesis

**Figure 1:**
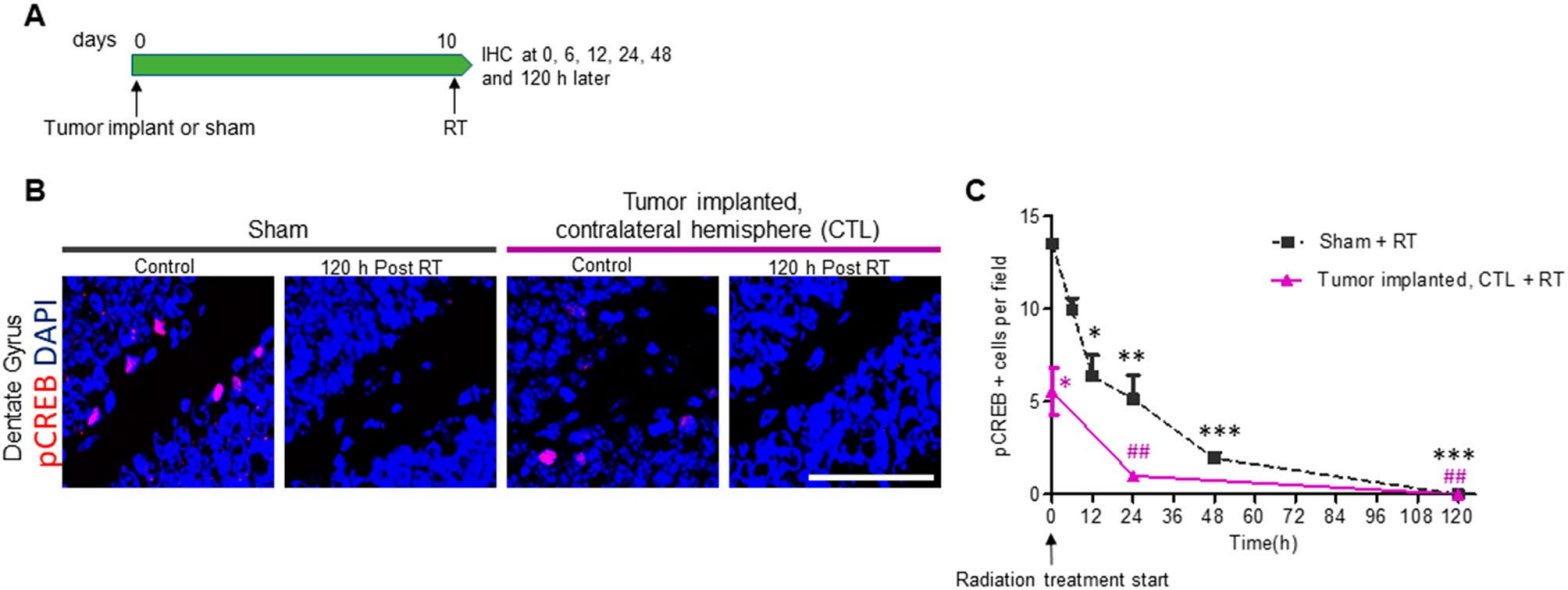
Radiation induced acute damage of neuroblasts in the subgranular zone (SGZ). (**A**) intracranial GL261 tumor implantation or sham operation was performed in C57BL/6 mice followed by 10 Gy cranial irradiation (RT) at 10 dpi. Mice were euthanized at 0, 6, 12, 24, 48 and 120 h after RT and brain samples processed for IHC. (**B**) Representative images of pCREB immunostaining at 0 (control) and 120 h after RT in sham and tumor implanted mice. Images were taken from the dentate gyrus (DG) of the hippocampus in the hemisphere contralateral (CTL) to the tumor implant site. Scale bar = 50 µm. (**C**) Quantification of pCREB positive cells in the SGZ at 0, 6, 12, 24, 48 and 120 h after RT. *n* = 3 per time point, *P < 0.05, **P < 0.01 and ***P < 0.001 compared to sham control and ^##^*P* < 0.01 compared to tumor implanted control mice by repeated ANOVA followed by Tukey’s post hoc *t* test.

Radiation at a dose higher than 2 Gy is known to induce apoptosis of neural precursor cells from the subgranular zone (SGZ) of the hippocampal dentate gyrus and cause almost complete long-term abolishment of the production of new neurons^12^. We sought to evaluate the time course of neural precursor cell dysfunction in the SGZ following radiation therapy (RT). To that end, we evaluated the activation of the transcription factor cAMP response element-binding protein (CREB), which plays a central role in the regulation of survival and development of new hippocampal neurons. Whole brain RT (10 Gy) was given to sham control and glioma-bearing mice at 10 days post implantation (dpi), and they were euthanized 0, 6, 12, 24, 48 and 120 h later (Fig. 1A). Activation of CREB was evaluated in the SGZ by immunostaining for phosphorylated-CREB (pCREB) in coronal sections of the hippocampus. By 6 h post RT in sham mice, the levels of pCREB decreased 26% and by 12 h post RT, the decrease was 53% (Fig. 1C,P < 0.05) in the SGZ. This decrease was greater (62% decrease) by 24 h (Fig. 1C,P < 0.01) and by 48 h there was an 86% decrease (Fig. 1C, P < 0.001). At 120 h after RT, no pCREB positive cells were detected in the SGZ (Fig. 1B,C, P < 0.001). The number of pCREB positive cells was already reduced (59% decrease) in glioma-bearing mice before RT compared to sham control mice (Fig. 1B,C,P < 0.05). At 24 h after RT there was an 82% decrease in pCREB positive cells (Fig. 1C, P < 0.01) and by 120 h, no positive cells were detected (Fig. 1B,C, P < 0.01). These acute changes suggest that radiation rapidly ablated neurogenesis in the hippocampus and intervention strategies aimed to protect hippocampal neurogenesis should be administered prior or concomitantly with radiation therapy.

### Glioma bearing mice that received combination of radiation therapy and PD-1 checkpoint blockade show persistent microglia activation in the subcortical white matter

**Figure 2:**
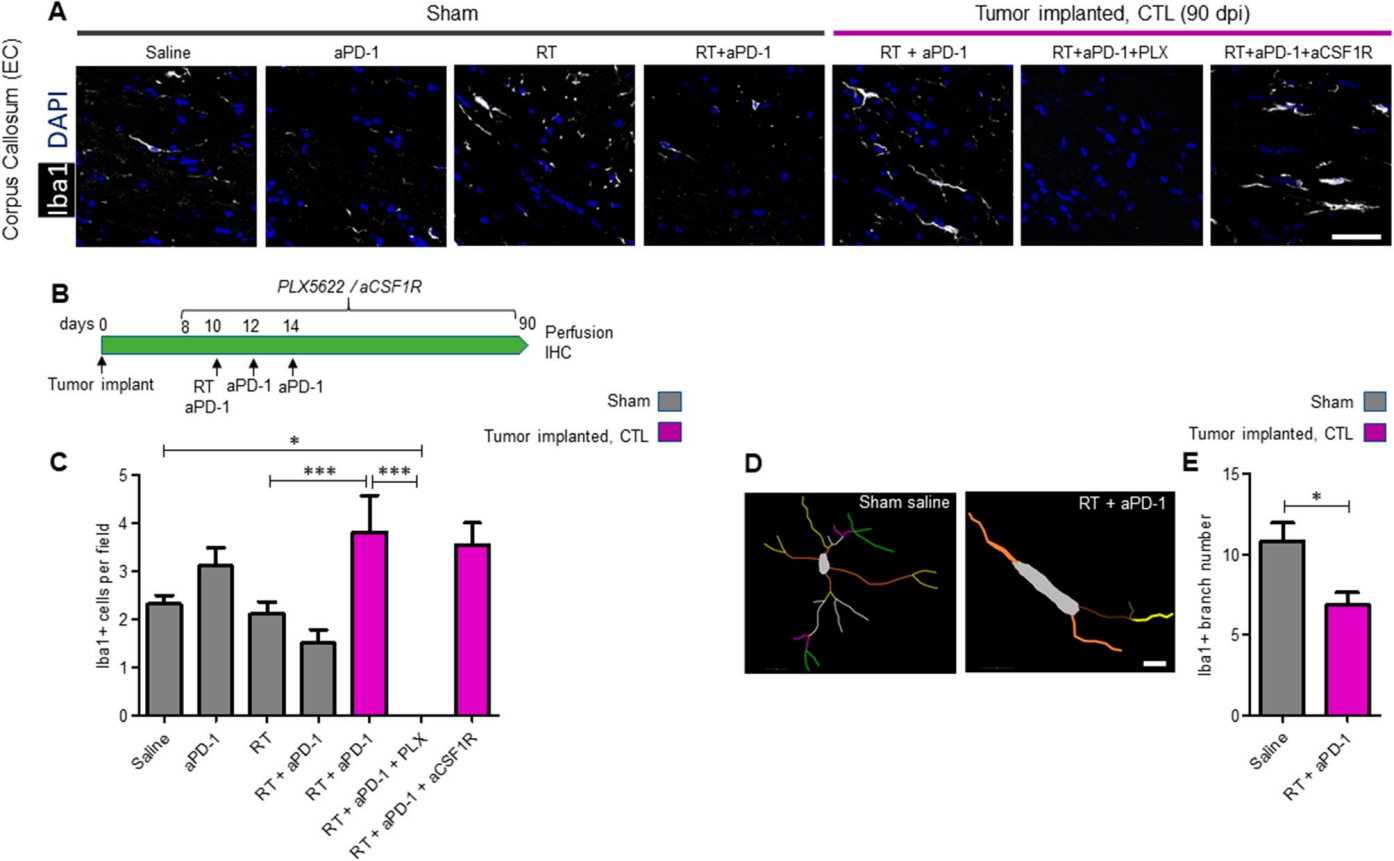
Tumor implanted mice treated with radiation in combination with aPD-1 showed chronic activation of microglia. (**A**) Sham operated mice were treated with either saline, aPD-1, RT or combined RT + aPD-1. Tumor implanted mice were treated with RT + aPD-1 plus either saline, PLX5622 or anti-CSF1R. Samples were collected at 90 dpi. Representative images of Iba1 IHC stain in the external capsule of the corpus callosum (EC) from the hemisphere contralateral to the implantation site (CTL) are shown. Scale bar = 50 µm. (**B**) Schematic of the experimental design for microglia elimination with PLX5622 and blockade of peripheral macrophages with anti-CSF1R blocking antibody in tumor implanted mice treated with RT + aPD-1. (**C**) Quantification of the numbers of Iba-1 cells in the EC of sham and tumor implanted mice. *n* = 6 per treatment arm in sham mice and *n* = 6 RT + aPD-1 + saline, *n* = 3 RT + aPD-1 + PLX5622 and *n* = 3 RT + aPD-1 + aCSF1R in tumor implanted mice, *P < 0.05, ***P < 0.001 by ANOVA followed by Tukey’s post hoc *t* test. (**D**) Fluorescent images of Iba1 + cells from sham mice and RT + aPD-1 treated tumor implanted mice were traced in Neurolucida software (https://www.mbfbioscience.com/neurolucida). Scale bar = 10 µm. (**E**) 3D reconstructions from the tracings were analyzed using the linear Sholl analysis method. The total number of branches per cell was significantly reduced in RT + aPD-1 treated tumor implanted mice. *n* = 3 per group, *P < 0.05 by Student’s *t*-test.

In our previous study, we established that the combination of RT and aPD-1 treatment achieved long-term survival in 75% of the mice implanted with a murine glioma cell line GL261^7^. At day 90, the mice that received RT + aPD-1 treatment were euthanized. Complete tumor regression was confirmed by microscopic analysis of coronal sections throughout the entire brain. Since glioma-bearing mice that receive either RT or aPD-1 as monotherapy do not survive more than 60 days, non-tumor implanted sham mice were used to evaluate the long-term effects of monotherapy versus combined therapy. Iba-1-immunostaining was used to identify microglia/macrophages in the brain hemisphere contralateral to the site of the tumor implantation (CTL). Cranial irradiation and anti-PD-1 treatment either as monotherapy or combination therapy did not produce changes in the number of Iba-1 positive cells in sham mice (Fig. 2A,C). On the other hand, tumor implanted mice that received RT + aPD-1 treatment showed 1.6-fold increase in the number of Iba-1 positive cells per 40X magnification field in the subcortical white matter compared to sham mice, suggesting activation of microglia or macrophages by the combined treatment (Fig. 2A,C, P < 0.05). We also observed an increase in Iba-1 positive cell numbers in the hippocampus (data not shown).

We next sought to identify whether the activated Iba-1 positive cells in the normal brain tissue of RT + aPD-1 treated mice are microglia or infiltrating bone marrow-derived macrophages. To this end, peripheral macrophages were depleted with intraperitoneal administration of anti-CSF1R (aCSF1R) blocking antibody, and microglia was depleted with PLX5622 treatment. Treatments with both cell blockers were initiated 48 h before initiation of the combined RT + aPD-1 therapy in glioma-bearing mice and continued until termination of the study (Fig. 2B). Blockade of macrophage infiltration with aCSF1R blockade treatment did not reduce the number of Iba-1 positive cells, however, PLX5622 treatment completely eliminated Iba-1 positive cells (Fig. 2A,C, P < 0.001) suggesting that microglia and not peripheral macrophages mediate long-term inflammation after RT + aPD-1 treatment in glioma-implanted mice. We analyzed the morphology of Iba-1 positive cells using high power confocal micrographs. Under pathological conditions, these cells undergo “activation” accompanied by morphological changes including the retraction of their processes and increase in cell body size^13^. Iba-1 positive cells from sham mice display a complex branching structure. However, microglia from RT + aPD-1 treated mice displayed an activated amoeboid phenotype with reduced number of processes compared to sham mice (Table 1 and Fig. 2D,E, P < 0.05).

**Table 1:** Summary of microglia characteristics in RT + aPD-1 treated mice and controls.

Measure	Sham saline (mean ± SEM)	RT + aPD-1 (mean ± SEM)	t-statistic	*P* value	Significance
Soma surface area (µm^2^)	180.76 ± 19.73	212.29 ± 20.73	1.101	0.286963	NS
Number of primary branches	3.38 ± 0.38	3.54 ± 0.31	0.335	0.742064	NS
Number of secondary branches	6.00 ± 0.93	4.31 ± 0.67	1.478	0.161516	NS
Branch endpoints	13.00 ± 1.45	6.85 ± 0.78	3.739	0.003852	**
Total branch length (µm)	244.39 ± 28.43	176.57 ± 22.71	1.863	0.085135	NS

The first column lists the measures of characteristics evaluated and the measurement units in parenthesis, when applicable. The second the third columns list the mean ± SEM for each measurement from the control group (sham saline) and RT + aPD-1 treated mice, respectively. The fourth column lists the t statistic for each measurement. The last column indicates the corresponding *P* value for Student’s *t* test. Microglia from RT + aPD-1 treated mice had fewer branch endpoints compared to the controls. Microglia characteristics were similar with regards to soma surface area, number of primary branches, number of secondary branches, and total branch length between the two groups.

**P < 0.01.

### Long-term depletion of subgranular zone neurogenesis and reduced subventricular zone neurogenesis in mice treated with RT + aPD-1

**Figure 3:**
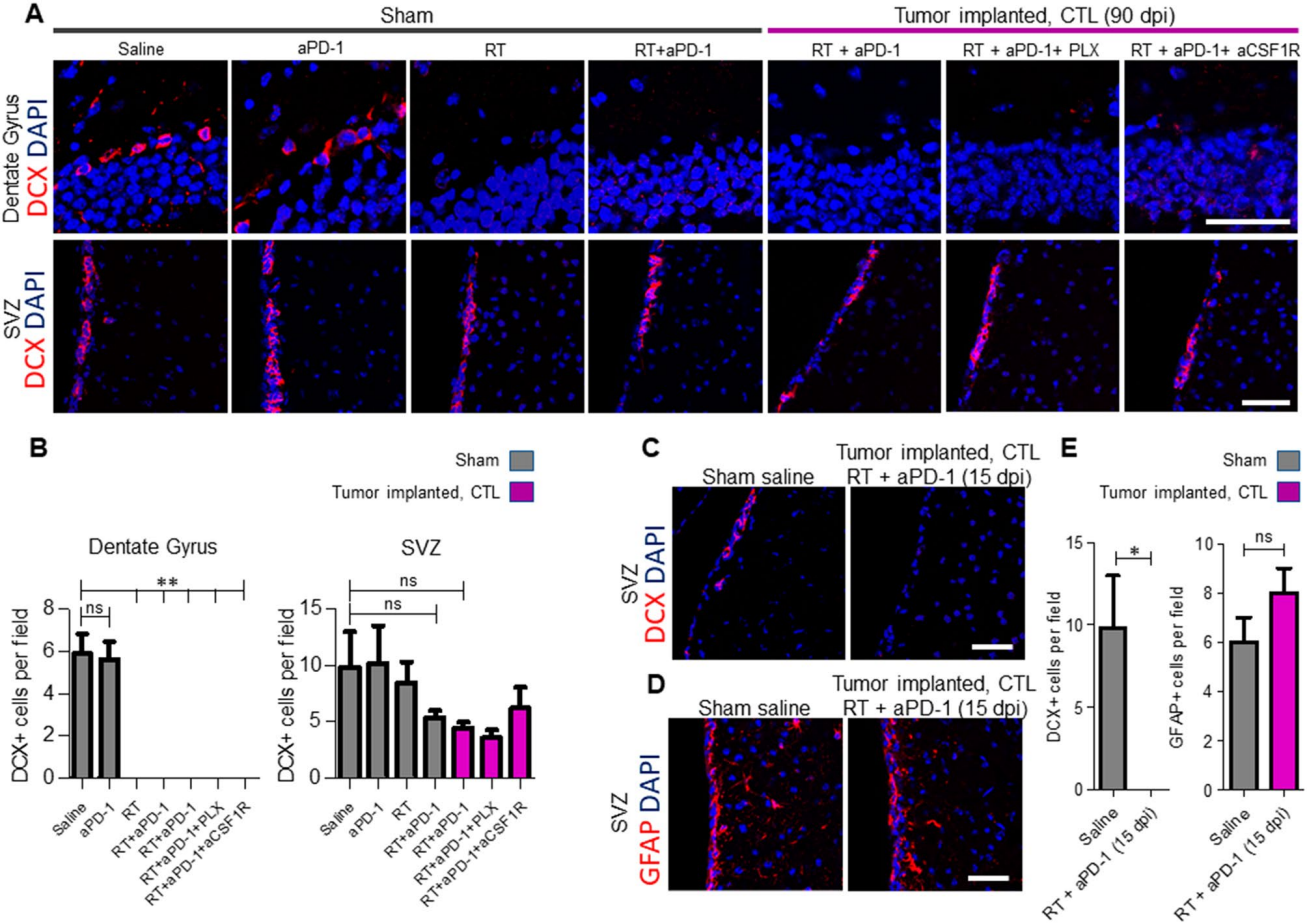
Long-term impact of whole-brain irradiations on neurogenesis in the subventricular zone (SVZ) and subgranular zone (SGZ). (**A**) Representative confocal micrographs of the dentate gyrus and the lateral ventricle stained for DCX in sham operated mice and tumor implanted mice for the indicated experimental groups. Samples were collected at 90 dpi and images taken from the contralateral hemisphere (CTL). Scale bar = 50 µm. (**B**) Quantification of DCX + cell number at 90 dpi in the SVZ and SGZ. *n* = 6 per treatment arm in sham mice and *n* = 6 RT + aPD-1 + saline, *n* = 3 RT + aPD-1 + PLX5622 and *n* = 3 RT + aPD-1 + aCSF1R in tumor implanted mice, **P < 0.01 by ANOVA followed by Tukey’s post hoc *t*-test. (**C**,**D**) Representative coronal sections of the lateral ventricle stained for DCX and GFAP respectively from saline treated shams and an RT + aPD-1 treated tumor implanted mice at 15 dpi. Scale bar = 50 µm. (**E**) Quantification of DCX + and GFAP + cell number in the SVZ at 15 dpi for the experimental groups in panel C and D. *n* = 6 per group, *P < 0.05 by Student’s *t* test.

We assessed the activity of neurogenesis in the hippocampus by staining for doublecortin (DCX), which is a marker of active cell division in neuronal progenitor cells. Specifically, we examined the loss of DCX-expressing neuroblasts in the SGZ of the dentate gyrus at day 90. Anti PD-1 blockade treatment alone in sham mice did not affect the number of DCX + neuroblasts in the SGZ (Fig. 3A,B). In contrast, sham mice that received RT or RT + aPD-1 showed complete ablation of DCX + neuroblasts, indicating that this effect was due to radiation (Fig. 3A,B, P < 0.01). Tumor-implanted mice that received RT + aPD-1 treatment also showed permanent depletion of DCX + neuroblasts in the SGZ which was not prevented by elimination of microglia by PLX5622 or by aCSF1R (Fig. 3A,B, P < 0.01). In constrast, we found that the number of DCX + neuroblasts in the contralateral subventricular zone (SVZ) at day 90 was not affected by treatments (Fig. 3A,B). To evaluate the early effects of treatment in the population of DCX + neuroblasts in the SVZ, we euthanized glioma-bearing mice at 5 days after initiation of RT + aPD-1 treatment (15 dpi). At this time point, we observed complete depletion of DCX + cells in the SVZ (Fig. 3C,E, P < 0.05). On the other hand, there was no change in the number of GFAP + type B neural stem cells (NSC) located within 30 µm of the lateral wall of the SVZ (Fig. 3D,E). These results suggest that the NSC were resilient to RT and have the capacity to regenerate the SVZ niche at later time points.

**Figure 4:**
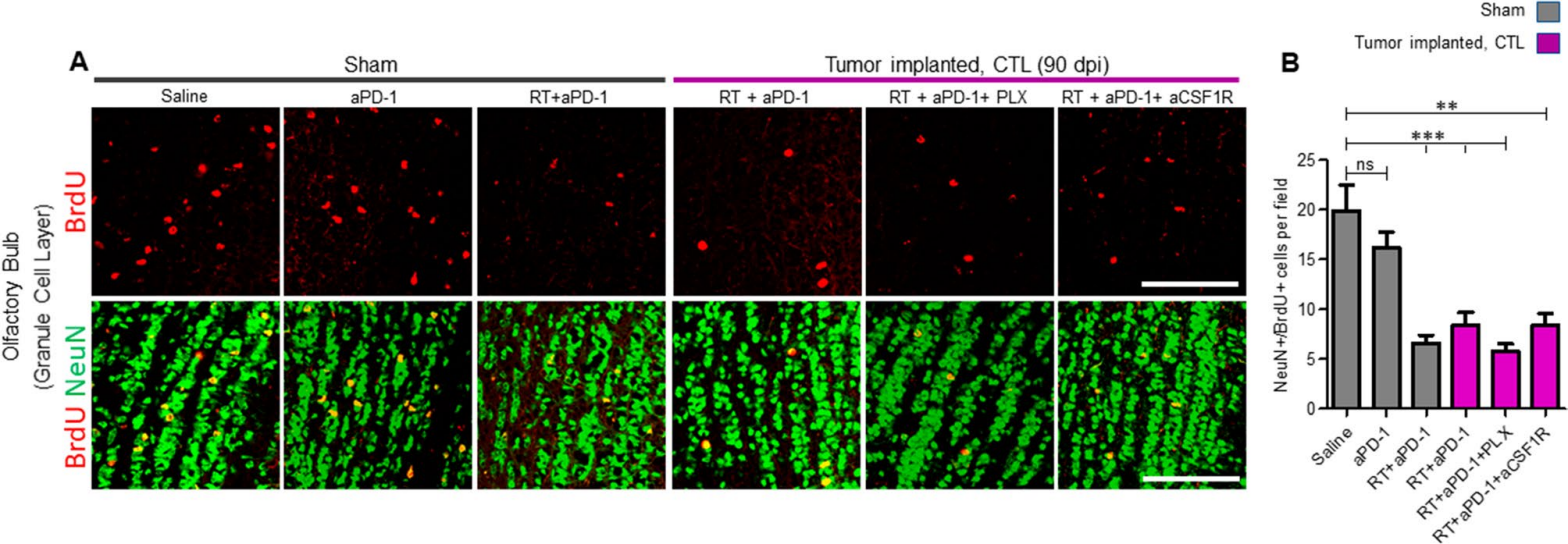
Treatment with RT + aPD-1 decreased the number of adult-born granule cells in the olfactory bulb. (**A**) Sham operated and tumor implanted mice received daily i.p. injections of BrdU starting at 60 dpi until 67 dpi. Coronal sections of the olfactory bulb (OB) from the hemisphere contralateral to the tumor implantation site (CTL) were collected at 90 dpi. Representative images from the granule cell layer (GCL) immunostained with antibodies against BrdU (red) and the neuronal marker NeuN (green) for the indicated experimental groups are shown. Scale bar = 100 µm. (**B**) Quantification of the number of adult-born granule cells (BrdU/NeuN double positive cells) in the GCL of the OB. *n* = 3 per group, **P < 0.01 and ***P < 0.01 by ANOVA followed by Tukey’s post hoc *t* test.

We further investigated the ability of these neuroblasts that repopulated the SVZ after RT + aPD-1 treatment to migrate and differentiate into neurons. In the adult mammalian brain, including humans, the neuroblasts from the SVZ migrate tangentially through the rostral migratory stream to the olfactory bulb (OB) where they differentiate into mature neurons in the granular cell layer (GCL)^14^. The mice were injected with BrdU for seven days (starting on day 60) and the OB samples collected on day 90. We quantified the number of new adult-born granular cells in the GCL assessed by double immunoreactivity of neuronal marker, NeuN, and proliferation marker, BrdU. Anti PD-1 checkpoint treatment alone in sham mice did not change the number of BrdU/NeuN double labeled cells (Fig. 4A,B). However, there was a 67% decrease in BrdU/NeuN double positive cells in sham mice that received RT + aPD-1 treatment compared to saline-treated sham mice (Fig. 4A,B, P < 0.001). Similarly, tumor-implanted mice that received RT + aPD-1 treatment showed a 58% decrease in BrdU/NeuN positive cells compared to sham saline mice (Fig. 4A,B, P < 0.001). Elimination of microglia or peripheral macrophages did not restore the production of OB neurons after RT + aPD-1 treatment (Fig. 4A,B).

### Loss of mature oligodendrocytes in the subcortical white matter is prevented by microglia elimination

**Figure 5:**
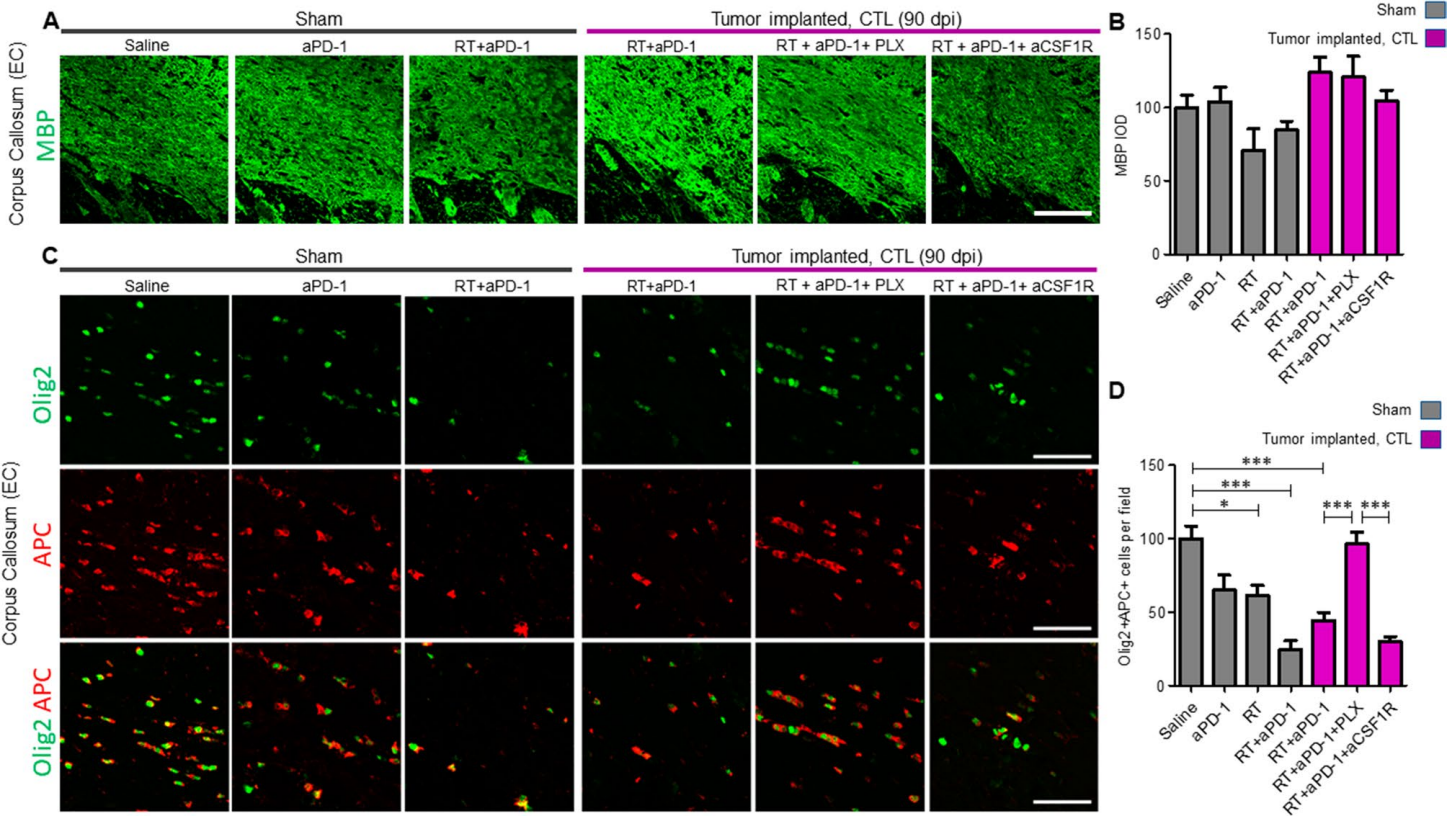
Microglia elimination prevented the loss of mature oligodendrocyte cells after RT + aPD-1 treatment. (**A**) Representative confocal micrographs of the external capsule of the corpus callosum (EC) stained for MBP in sham operated mice and tumor implanted mice for the indicated experimental groups. Samples were collected at 90 dpi and images taken from the contralateral hemisphere (CTL). Scale bar = 50 µm. (**B**) Quantification of MBP IOD in the EC. (**C**) Confocal images of the EC immunostained for Olig2 and APC for the experimental groups in panel A. Colocalization of both markers is also shown. (**D**) Quantification of mature oligodendrocytes assessed by Olig2 and APC double immunoreactivity in the EC. *n* = 6 per treatment arm in sham mice and *n* = 6 RT + aPD-1 + saline, *n* = 3 RT + aPD-1 + PLX5622 and *n* = 3 RT + aPD-1 + aCSF1R in tumor implanted mice, *P < 0.05 and ***P < 0.001 by ANOVA followed by Tukey’s multiple comparison test.

**Figure 6:**
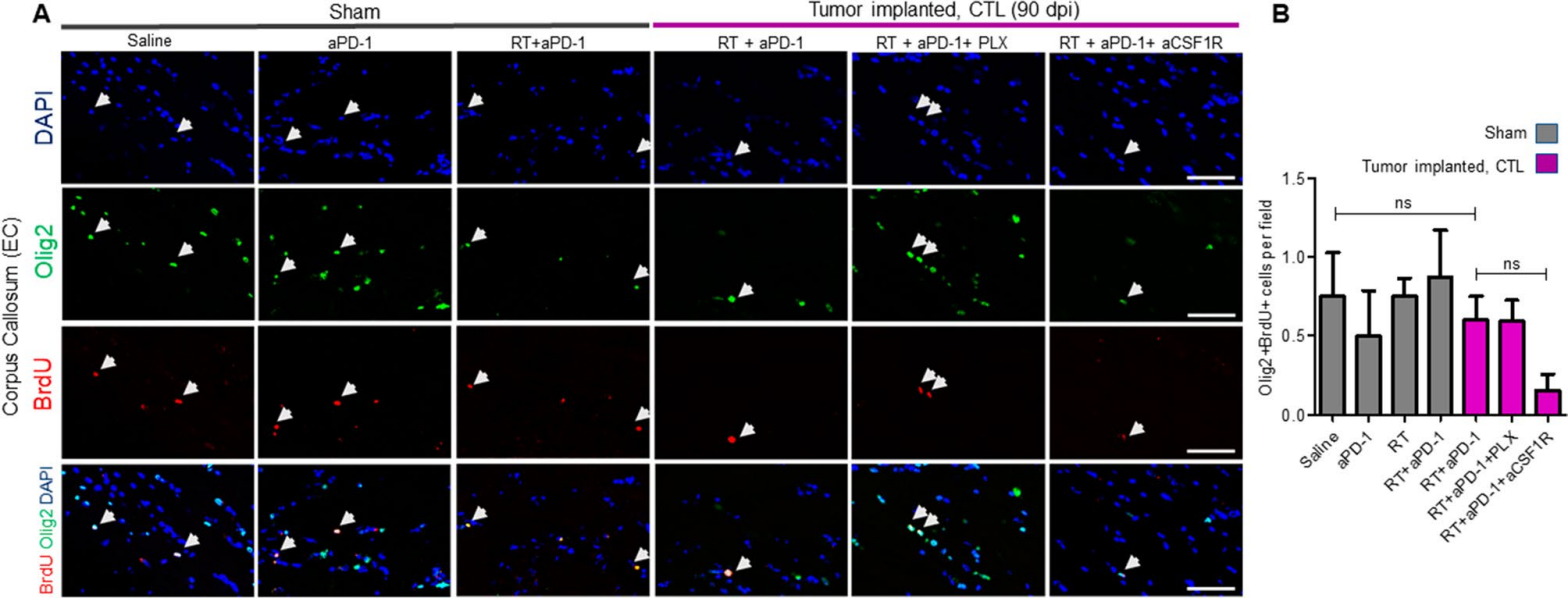
Proliferation of oligodendrocyte precursors is not changed by RT + aPD-1. (**A**) Sham operated and tumor implanted mice received daily i.p. injections of BrdU starting at 60 dpi until 67 dpi. Brain coronal sections were collected at 90 dpi and immunostained with specific antibodies for BrdU (red) and the oligodendrocyte marker Olig2 (green). Representative micrographs of the external capsule of the corpus callosum (EC) for the indicated experimental groups are shown. Images were taken from the contralateral hemisphere (CTL). Arrows indicate double positive cells, scale bar = 50 µm. (**B**) The number of newly generated oligodendrocytes assessed by Olig2 and BrdU double immunoreactivity was quantified in the EC. *n* = 3 per group.

Several lines of evidence indicate that radiation-induced inflammation results in white matter damage^15–17^. We used MBP immunostaining to evaluate the subcortical white matter in the different mouse groups. We did not find marked demyelination or differences in the intensity of MBP immunoreactivity in any of the treatment groups (Fig. 5A,B). Since anti-MBP immunostaining is not always sensitive enough to visualize changes that occur in the white matter, we evaluated the number of mature oligodendrocytes by APC and Olig2 double immunostaining. Treatment with RT alone showed a mild reduction (39% decrease) of the number of oligodendrocytes in sham mice (Fig. 5C,D, P < 0.05). Interestingly, the combination of RT + aPD-1 in sham mice resulted in a more drastic reduction (75% decrease, *P* < 0.001) of Olig2 + /APC + cells compared to saline treatment (Fig. 5C,D, P < 0.001). Tumor-implanted mice treated with RT + aPD-1 also showed a significant reduction (56% decrease) of oligodendrocytes compared to control sham saline mice (Fig. 5C,D, P < 0.001). Strikingly, the number of oligodendrocytes following RT + aPD-1 in glioma-implanted mice was restored to normal levels by microglia elimination (Fig. 5C,D). On the other hand, peripheral blockade of macrophages did not have any effects (Fig. 5C,D). Impressed with the extent of recovery in the number of oligodendrocytes achieved by microglia elimination, we investigated whether PLX5622 treatment increased the proliferation of oligodendrocyte precursor cells (OPCs) in the periventricular white matter. The proliferation of OPCs assessed by BrdU and Olig2 double immunoreactivity was similar among the different experimental groups (Fig. 6A,B). Microglia elimination with PLX5622 treatment did not produce any changes in the number of BrdU/Olig2 double positive cells (Fig. 6A,B). Macrophage depletion resulted in a trend toward reduced BrdU/Olig2 double positive cells, although this decrease was not significant (Fig. 6A,B. *P* > 0.05).

## Discussion

Immunotherapy offers a promising therapeutic option for brain cancers. There are currently close to twenty ongoing clinical trials evaluating the use of anti PD-1 checkpoint blockade in combination with radiation therapy^28^, however the effects of this combination on the normal brain tissue have not been investigated in detail. Of the recently completed studies, two sister trials, CheckMate-498^29^ and CheckMate-548^30^, evaluated the addition of PD-1 inhibitor Nivolumab to the adjuvant chemoradiation for glioblastoma with unmethylated and methylated MGMT promoter, respectively. Final publications of their findings are still pending, but the preliminary press releases from Bristol-Myers indicate these studies have failed to demonstrate survival advantages to the addition of immunotherapy to the standard adjuvant regimen. The reasons for why these trials have failed, despite such promising data in preclinical models, can be multiple and will likely be elucidated as the numerous ongoing trials start reporting their results. For example, it should be noted that in both CheckMate-498 and CheckMate-548 fully fractionated radiation therapy to a total dose of 60 Gy in 2 Gy per fraction was used. In contrast, a number of reports suggest that regimens, utilizing higher dose per fraction, as in stereotactic radiotherapy, rather than the conventional fully fractionated radiation schedules, may be uniquely immunogenic when combined with checkpoint inhibitors^31,32^. Interestingly, fractionated stereotactic radiotherapy, utilizing 8 to 10 Gy per fraction (i.e. the same dose per fraction range that was found to be “immunogenic” in the preclinical studies by Demaria and colleagues) also showed some promise in prior clinical investigations for patients with recurrent glioblastoma^33^.

None of the currently reported studies of combining radiotherapy and immunotherapy for glioblastoma have thus far provided detailed evaluations of the normal tissue toxicity, such as neurocognitive sequelae, of the combined treatment. The CheckMate-143 clinical trial, comparing Nivolumab to Bevacizumab for recurrent glioblastoma, has reported similar toxicities for the two arms, with a somewhat higher absolute number of neurologic adverse events in the Nivolumab cohort, not reaching statistical significance. However, this study did not examine the combination of radiotherapy and immunotherapy, nor did it study any long-term sequelae of the immunotherapeutic treatment in the brain. Thus, the question which we seek to answer in the current investigation has thus far not been addressed in any of the ongoing or recently completed clinical trials. In the preclinical setting, a recent study characterized the inflammation-induced neurological dysfunction by radiotherapy to tumor bearing hind flank of mice in combination with anti-CTLA4 blockade in preclinical models^18^. However, brain tumor patients are exposed to direct damaging effects to the brain induced by RT, surgical procedures, and vascular damage caused by the tumor such as hemorrhage and ischemia, all of which contribute to the resulting neurological dysfunction. The mouse brain tumor model used in this study together with the treatment efficacy of RT + aPD-1 combination provides a great tool to evaluate the long-term adverse effects to the normal brain in animals that achieved complete tumor regression.

In our previously published study, we showed that RT + aPD-1 combined treatment induced the activation of CD8 T cells and the proinflammatory polarization of macrophages and microglia in the tumor microenvironment^7^. At the long-term time point evaluated in the present study, we did not find CD8 + T cells in the normal brain tissue. However, there was an increase in the number of microglia cells with an activated phenotype in the subcortical white matter and hippocampus of mice that achieved complete tumor regression after RT + aPD-1 treatment. This effect appears to be related to the previous presence of the tumor since sham mice treated with RT + aPD-1 did not show a chronic increase in the number of microglia. The mechanism of chronic microglia activation in tumor-bearing RT + aPD-1 treated mice remains to be fully understood. One possibility is that it is sustained by proinflammatory cytokines released by the tumor after RT + aPD-1 treatment. For instance, high levels of interferon gamma, which is regarded as a potent signal for microglia activation^19,20^, were expressed by tumor-infiltrating CD-8 T cells up to 20 days post treatment^7^.

Radiation-induced inflammation has been proposed as a mechanism that contributes to impairment in hippocampal neurogenesis and cognitive decline after cranial irradiation. Limoli et al.^5^ found that microglia elimination improved hippocampal-dependent learning and memory function after 9 Gy whole brain radiation. Our results showed that all mice treated with RT alone or combined with aPD-1 have permanent depletion of neuroblasts in the subgranular zone (SGZ) of the hippocampal dentate gyrus. Although microglia elimination did not protect SGZ neuroblasts, it could protect hippocampal function after RT + aPD-1 by other mechanisms. For instance, it was shown that long-term elimination of microglia in models of brain inflammation resulted in the restoration of the number synapses in the CA1 region of the hippocampus^21^. Further studies will look at the ultimate hippocampal memory function and the structural changes of synapses in mice after RT + aPD-1 for gliomas as well as the role of microglia activation in those changes. Of note, we found that neuroblast survival in the dentate gyrus of glioma bearing mice was already reduced prior to treatment. It is possible that tumor growth resulted in the compression and displacement of the contralateral hippocampus affecting neurogenesis. This is consistent with the findings of randomized phase III trials where the majority of patients with brain tumors presented evidence of cognitive impairment at baseline that was correlated with the lesion volume^22^. In contrast with the neuroblasts depletion in the SGZ, there is a repopulation of the subventricular zone (SVZ) neuroblasts pool after RT. It is plausible that GFAP expressing type B neural stem cells (NSCs) survived and divided to restore DCX-positive neuroblasts. In fact, when we looked at an earlier time point after RT, we found depletion of DCX + neuroblasts but normal numbers of GFAP positive cells. These findings are in agreement with the study of Cameron et al.^23^ who found that compared to neuroblasts, type B NSCs express higher levels of the antiapoptotic proteins Bcl2 and Mcl1 and are resilient to a combination of RT and Temozolomide. Regardless of the recovery in the number of SVZ neuroblasts after RT + aPD-1, these newly generated neuroblasts have a reduced ability to migrate and differentiate into mature neurons. Studies in animal models have shown that SVZ neurogenesis is activated after different forms of brain injury such as stroke or traumatic brain injury and it favors a recovery in motor function^24^. Since it was reported that cranial irradiation results in up to 100-fold increase in the risk of stroke due to delayed vasculopathy^25^, a limited capacity of SVZ-neurogenesis may have negative consequences in patients that become long-term survivors after RT + aPD-1 treatment.

Our study identifies a pattern of normal tissue changes in cured mice after the combined RT + aPD1 therapy for glioma, i.e. the loss of mature oligodendrocytes in the subcortical white matter. These changes were not observed in the monotherapy arms, indicating the interplay between radiation and immunotherapy. Furthermore, we observed that a depletion of microglia completely blocked RT + aPD-1 mediated loss of mature oligodendrocytes, indicating that microglia were required as mediators of white matter injury following treatment. Given that 10 Gy RT directly induces apoptosis of oligodendrocytes within 24 h after treatment^26^, our results could be explained by the “two-hit” model proposed by Marzan et al.^27^ to explain the role of activated microglia in the demyelination induced by cuprizone. Applying to the model, RT would damage oligodendrocytes rendering them sensitive to microglia-mediated induction of apoptosis. But in the absence of microglia, the damaged oligodendrocytes which are still viable may survive. Increased microglia activation following RT + aPD-1 seems to correlate with the drastic decrease in the number of oligodendrocytes.

Because there were no long-term survivors in the glioma-bearing groups treated with RT alone or anti-PD-1 alone, our analysis has been constrained by having to compare the long term effects of the RT + anti-PD-1 dual therapy for glioma to the corresponding effects of single modality treatments (RT or anti-PD-1) in non-tumor bearing mice. We recognize this approach to have clear limitations, as the immune changes observed post-treatment in the normal brain may be different than those seen in a brain affected by tumor (even after the tumor has been cured). Ultimately, in the clinical setting the most important question is what happens to normal brain tissue after the multimodality treatment of glioma, and to answer this question we must examine what happens in tumor-bearing mice who become long-term survivors of their disease. However, to effectively parse between the normal tissue effects of treatment versus those of the disease itself, it would be important to also compare the single modality therapies to the combined therapy in non-tumor bearing mice. This comparison should certainly be included in future investigations aimed at further delineating the normal tissue effects of glioma-directed therapies.

To the best of our knowledge, this is the first preclinical study to evaluate the long-term neuroinflammatory effect of the normal brain tissue after RT + aPD-1 combined treatment in the normal brain tissue uniquely in the mice cured from the glioma. These results support the consideration of microglia elimination as a therapeutic strategy to mitigate white matter toxicity in patients treated with RT + aPD-1 for brain tumors. The ability of PLX5622 to cross the blood–brain barrier and rapidly and efficiently deplete microglia makes it a potentially promising drug.
